# Inconsistent estimates of forest cover change in China between 2000 and 2013 from multiple datasets: differences in parameters, spatial resolution, and definitions

**DOI:** 10.1038/s41598-017-07732-5

**Published:** 2017-08-18

**Authors:** Yan Li, Damien Sulla-Menashe, Safa Motesharrei, Xiao-Peng Song, Eugenia Kalnay, Qing Ying, Shuangcheng Li, Zongwen Ma

**Affiliations:** 1 Department of Atmospheric and Oceanic Science, University of Maryland, College Park, Maryland 20742 USA; 2 The Institute for Physical Science and Technology, University of Maryland, College Park, Maryland 20742 USA; 3 College of Urban and Environmental Sciences, Peking University, Beijing, 100871 China; 4 Department of Earth and Environment, Boston University, Boston, Massachusetts 02215 USA; 5 Department of Physics, University of Maryland, College Park, Maryland 20742 USA; 6 Department of Geographical Sciences, University of Maryland, College Park, Maryland 20742 USA; 7 Key Laboratory for Earth Surface Processes of The Ministry of Education, Peking University, Beijing, 100871 China; 8 China Science and Technology Exchange Center, Beijing, 100045 China

## Abstract

The Chinese National Forest Inventory (NFI) has reported increased forest coverage in China since 2000, however, the new satellite-based dataset Global Forest Change (GFC) finds decreased forest coverage. In this study, four satellite datasets are used to investigate this discrepancy in forest cover change estimates in China between 2000 and 2013: forest cover change estimated from MODIS Normalized Burn Ratio (NBR), existing MODIS Land Cover (LC) and Vegetation Continuous Fields (VCF) products, and the Landsat-based GFC. Among these satellite datasets, forest loss shows much better agreement in terms of total change area and spatial pattern than do forest gain. The net changes in forest cover as a proportion of China’s land area varied widely from increases of 1.56% in NBR, 1.93% in VCF, and 3.40% in LC to a decline of −0.40% in GFC. The magnitude of net forest increase derived from MODIS datasets (1.56–3.40%) is lower than that reported in NFI (3.41%). Algorithm parameters, different spatial resolutions, and inconsistent forest definitions could be important sources of the discrepancies. Although several MODIS datasets support an overall forest increase in China, the direction and magnitude of net forest change is still unknown due to the large uncertainties in satellite-derived estimates.

## Introduction

Forest change has broad implications for climate^1–3^, ecology^4–6^, hydrology^1, 7^, and human wellbeing^8–10^. Numerous observational and modeling studies have shown that deforestation and afforestation have significant impacts on climate through their direct biophysical effects^2, 11–13^ and indirect effects on the carbon cycle^14–17^. Monitoring change in forest cover, therefore, is essential to understanding these impacts and is critical to forestry policy-making and management.

Forest change can be monitored either through national forest inventories or with remote sensing observations. The national forest inventories (NFIs), led by governmental organizations, provide systematic and accurate information of forest resources over a long time span. For example, in China, there have been eight NFIs implemented by the State Forest Administration^18^ with data collected from a large number of distributed permanent sample plots (4.1 × 10^5^ plots in the 7^th^ NFI^19^ but these plot-level data are not open to the public). However, NFI definitions of forest, sampling standards, and sampling methods vary by country and over time^16, 20, 21^, which could produce inconsistencies in the data.

Remote-sensing-based methods can provide timely data with high spatial and temporal resolutions to better capture forest dynamics such as degradation and forest gain^22–24^. Satellite data from the Moderate Resolution Imaging Spectroradiometer (MODIS) as well as from the Landsat Thematic Mapper (TM) and Enhanced Thematic Mapper Plus (ETM+) have been widely used to identify various types of forest changes including disturbance, loss, and regrowth^25–32^. MODIS data have high temporal resolution but relatively coarse spatial resolution (250 or 500 m). This resolution makes the data useful for regional and global-scale applications but limits their ability to detect finer-scale changes^32^. In contrast, the higher spatial resolution of Landsat data (30 m) is accompanied with large data volumes for which regional to global-scale applications have not been feasible until recent improvements in computational resources^22, 23^. The newly available Global Forest Change (GFC) product^33^ based on Landsat imagery is the first high-resolution dataset of this kind that offers global and annual forest cover change starting from year 2000.

The information from the NFI and from the remote sensing datasets should complement each other and could be combined to better understand forest dynamics (e.g., in estimating forest area^34, 35^ and biomass^36, 37^). However, in practice, this may not necessarily be the case for forest cover change^38, 39^ due to the different definitions and methodologies employed^40, 41^. For example, the NFI and GFC datasets show conflicting findings for China’s forest cover change during the 2000s (2000–2013). According to the Chinese NFI, national forest coverage has increased by 2.15% in the 7^th^ NFI (2004–2008) and by 3.41% in the 8^th^ NFI (2009–2013) when compared to the 6^th^ NFI (1999–2003). The increases in forest area have been attributed to the successful implementation of several afforestation and forest protection programs^21^, including the Natural Forest Conservation Program (NFCP)^42^, the “Three-North” Shelter forest program^43^, and the “Grain for Green” program^44, 45^. By contrast, the GFC dataset, which is considered to be more accurate than previous remote sensing datasets due to its unprecedented global high spatial resolution, showed that the change in forest area of China between 2000 and 2012 was a net loss of 38,743 km^2^, equivalent to a decline of 0.40% in national forest coverage (i.e., forest area divided by the total land area of China). This discrepancy could profoundly influence studies that rely on forest change data and represents an important source of uncertainty in evaluating the impacts of China’s afforestation efforts. For example, the forest increase in NFI has been frequently used to explain the vegetation greening trend observed in China over the past several decades^46, 47^, and its climate impacts have also attracted researchers’ attention^3, 48–51^. However, the conclusions of those studies could be potentially affected if the reported forest increase in NFI was not supported by other independent datasets.

In this study, we collected data from multiple sources and compared their estimates of forest cover change based on different methodologies to better understand how forest cover has changed in China, and to investigate potential sources of disagreement across these datasets. An ideal way to resolve the discrepancies in forest cover change would be to directly validate the change in each dataset using ground reference data and give an unbiased estimate^52^. However, this cannot be done currently due to the lack of ground reference data. In addition, it is very challenging to validate forest cover change estimates^52, 53^, especially over large regions^22^.

## Data and Methods

In this work, we used four satellite-derived datasets and one independent statistical forestry inventory dataset (Table 1). The satellite datasets included three MODIS-based datasets: (1) forest cover change produced by a new algorithm, MODTrendr (MODIS-based detection of Trends in Disturbance and Recovery), using the Normalized Burn Ratio (NBR)^29^; (2) the Collection 5 MODIS land cover product^54^; (3) the Collection 5 MODIS Vegetation Continuous Fields (VCF) product^55^; as well as (4) the Landsat-derived Global Forest Change product aggregated to 500 m spatial resolution. In addition to these satellite-derived datasets, we used (5) the Chinese Forestry Inventory (NFI) dataset, which is in the form of statistical data. It is worth mentioning that forest cover change estimates from MODIS NBR and GFC were mapped directly from spectral changes in time series of satellite imagery while those estimates from MODIS LC, VCF, and NFI were based on comparing forest map/statistics between different periods.

**Table 1:** Overview of different datasets employed for forest cover change estimates.

Name	Time span	Source	Spatial resolution	Data type^a^	Change detection method
MODIS NBR (based on MODTrendr)	2000–2013 Yearly	MODIS	500 m	Binary	Detect change from time series
MODIS LC	2001–2012 Yearly		500 m	Binary	Change in land cover type between two periods
MODIS VCF	2000–2013 Yearly		250 m	Numeric	Change in fractional tree cover between two periods
GFC	2000–2012 Yearly for loss and aggregated for gain	Landsat	30 m	Binary	Detect change from time series
NFI	1999–2013 Five-years interval	Statistics	Provincial	Numeric	Change in forest area between two periods

^a^Binary: Change (loss or gain) or no change. Numeric: Change represented in units of area.

### MODIS NBR based on MODTrendr

MODTrendr was developed based on the LandTrendr algorithm^30^, which uses time-series of Landsat reflectance data to detect forest change, and was adapted to work on MODIS surface reflectance data^29^. The algorithm produces a temporal segmentation of a reflectance time series that can be useful to characterize long-term dynamics in forest properties, including disturbance processes (abrupt or gradual) and vegetation growth and recovery^29, 30^. Here we applied the MODTrendr algorithm to an annual time series (2000 to 2013) of the Normalized Burn Ratio (NBR), a spectral index derived from peak-summer observations for each year from the MODIS Collection 5 Nadir BRDF-Adjusted Reflectance product (MCD43A4). NBR has been proven to be sensitive to various types of forest disturbance (e.g., fire^56^, insect^57^, and infrastructure construction^58^) and recovery after disturbance^56, 59^; thus it has been widely applied to the problem of forest change detection^58, 60^. Forest cover change (forest loss, gain, or no change) at each pixel can be identified from the segments of the NBR time series (i.e., the slope of the segments produced by MODTrendr, Supplementary Fig. S1). Some user-defined parameters need to be specified, including an NBR threshold to distinguish forest and non-forest, a threshold to distinguish forest cover change from no change, and an independent forest mask to filter out change signals that occur in non-forest land cover types (for more details see Supplementary Information). The resulting forest cover change estimates are referred to as “MODIS NBR” hereafter. One of the strengths of the MODTrendr algorithm is its flexibility; it can be applied to a time series of any spectral band or index at any spatial resolution. This feature allowed us to investigate the sensitivity of the results to parameter values that are critical to forest cover change detection would not be possible with other existing MODIS products.

### MODIS LC

The MODIS Collection 5 Land Cover (LC) product (MCD12Q1)^54^ provides annual land cover information from 2001 to 2012. The IGBP (International Geosphere-Biosphere Program) classification scheme provided by this product consists of 17 land cover types, including five forest classes: evergreen needleleaf, evergreen broadleaf, deciduous needleleaf, deciduous broadleaf, and mixed forests. These forest classes were grouped together to define the “forest” from the MODIS LC product as they all have woody vegetation with stature greater than 2 m and coverage greater than 60% at the MODIS pixel scale^61^. To minimize the artificial interannual variation in land cover types in the MODIS LC product^54^, we first created two stable land cover maps by using the most frequent land cover type at each pixel during two periods, 2001–2003 and 2010–2012. By comparing these two stable land cover maps, we defined forest loss as when a forest pixel in the first period was converted to a non-forest pixel in the later period. A change in the opposite direction was defined as forest gain. The land cover classification accuracy for a single year of MODIS LC is available in ref. 54, however, errors could be inflated by such a post-classification change detection method^53^.

### MODIS VCF

The Collection 5 MODIS Vegetation Continuous Field product (MOD44B)^55^ provides yearly tree cover percentage information at a global scale. The tree cover fraction was interpreted as the proportion of forested land in each MODIS pixel. Similar to our pre-processing of the MODIS LC dataset, we removed some inter-annual variation in tree cover that did not represent forest cover change^31^ by creating two maps of average tree cover percentage for two periods, 2000–2002 and 2011–2013. Forest gain and loss were defined as an increase or decrease in tree cover between these two periods, respectively. The accuracy of VCF data has been reported in ref. 62, but similar to MODIS LC, errors could be inflated by post-classification change detection.

### GFC data

The Global Forest Change (GFC) data were produced from Landsat ETM+ time series at 30 m spatial resolution^33^. These data provide a baseline tree cover percentage in 2000, as well as forest loss and gain labels (binary) for each pixel afterwards through 2000–2012. Forest in GFC was defined as all vegetation taller than 5 m in stature^33^. It should be noted that forest loss is provided for each year whereas forest gain is provided over the entire period. Therefore, forest loss can be accumulated through the period by labeling any pixels as “forest loss” if a loss occurred during any year (similar to the MODIS NBR results). An explicit accuracy assessment of forest loss and gain in GFC can be found in ref. 33.

### NFI data

The Chinese National Forest Inventory (NFI) dataset contains the official forestry statistics derived from extensive field surveys at five-year intervals, combined with remote sensing technology^63^. Each inventory was conducted over a 5-year period, but for each province the survey could be completed in different years within the 5-year span^18, 64^. Forest in NFI was defined as woodland with canopy coverage greater than 20%. More details about the techniques and standards of NFI can be found in refs 63 and 64. To better match the temporal coverage of satellite data, we used forest area statistics that were reported in the 6th (1999–2003) and 8th (2009–2013) NFI, available at national and provincial levels (http://211.167.243.162:8085/8/chengguobaogao/showpageinit?lm=xxxz). The provincial NFI data include forest area for 22 Provinces, 4 Municipalities, and 5 Autonomous Regions of China. Hong Kong, Macao, and Taiwan were not included in this analysis because they were not covered by NFI. Forest change was extracted from the difference in forested area between the two NFIs. The accuracy requirement of forest area in NFI was 95% for provinces with forest coverage more than 12% and was 90% for the remaining provinces^63^.

All of the satellite data were analyzed in the 500 m MODIS Sinusoidal projection (an equal-area projection). The 500 m spatial resolution was chosen for the analyses because it was the native resolution of two of the three MODIS-derived datasets and was also the coarsest of all datasets, which can avoid the uncertainty that would be otherwise introduced from downscaling the coarse data to higher resolution. The original 250 m VCF product was aggregated to 500 m by averaging each set of four 250 m pixels, and forest loss and gain in GFC were aggregated to percentage of change at the 500 m spatial resolution. These aggregations kept the meaning of the quantitative information of VCF and GFC (percentage tree cover) the same as in their original resolutions. The national total forest change area was calculated as the summed area of each 500 m forest change pixel for MODIS NBR and LC datasets, and it was the summed area of sub-pixel fractional change of each forest change pixel for MODIS VCF and GFC.

## Results

### Forest cover loss estimates from satellite datasets

**Figure 1:**
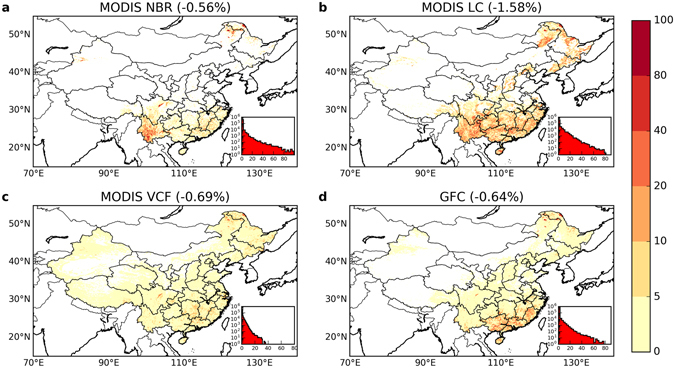
Spatial pattern in forest loss (%) in China between 2000 and 2013 from different satellite datasets. These patterns were computed from similar (but not identical) time periods: (**a**) MODIS NBR for 2000–2013, (**b**) MODIS LC for 2001–2012, (**c**) MODIS VCF for 2000–2013, (**d**) Landsat GFC for 2000–2012. The total forest loss area was 54,019 km^2^ in MODIS NBR, 151,846 km^2^ in MODIS LC, 65,948 km^2^ in MODIS VCF, and 61,165 km^2^ in GFC. The forest change results were aggregated to percentage at 5 km resolution for display purposes. The histogram in the inset (lower right of each panel) shows the corresponding count of 5 km pixels binned into categories of discrete forest loss percentages. The number in parenthesis after the title for each panel shows the corresponding change in national forest coverage based on the country area of 9.6 million km^2^. Maps were created using Python 2.7.11 (https://www.python.org/).

**Figure 2:**
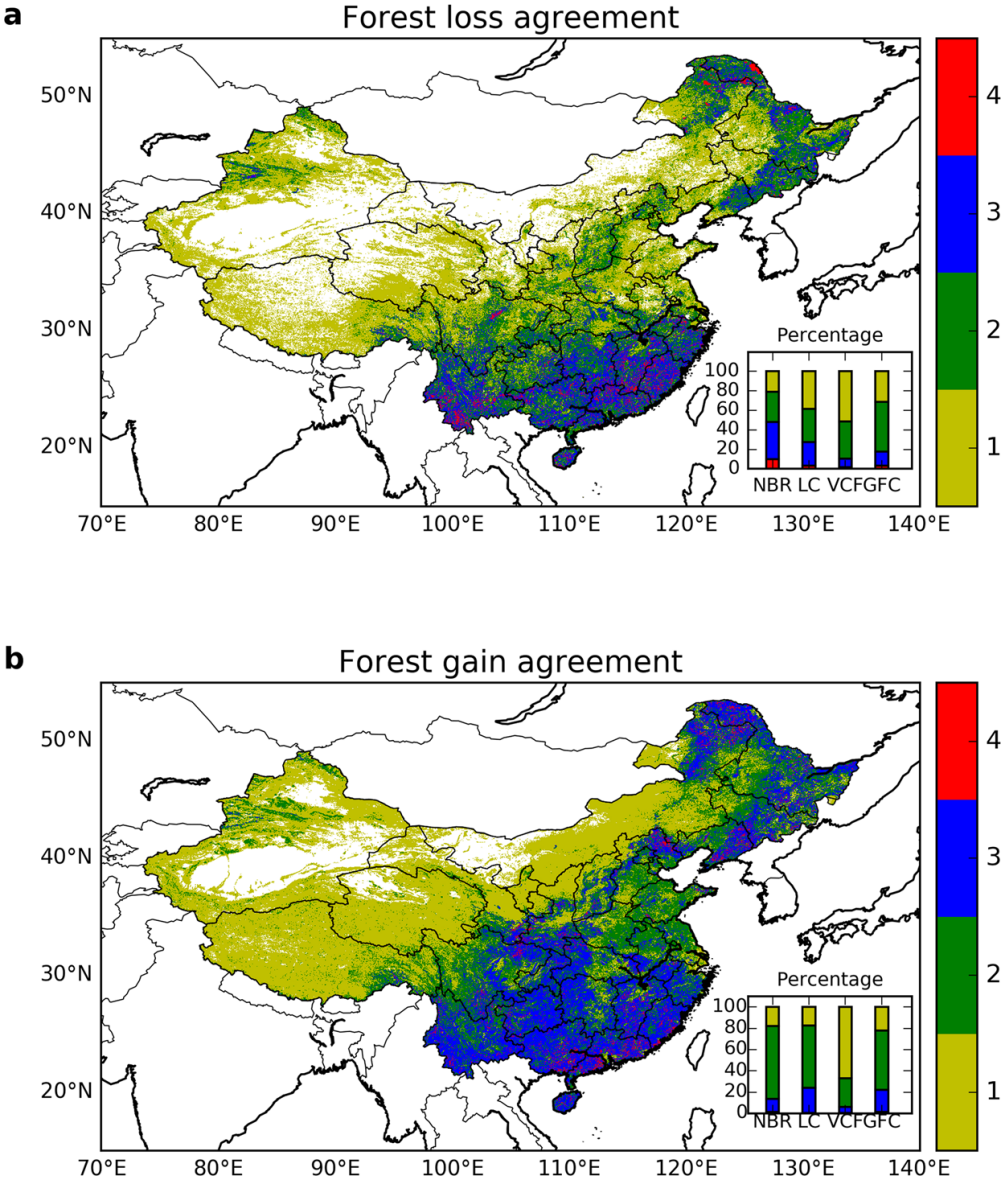
Forest cover change agreement among four satellite datasets: (**a**) Forest loss agreement and (**b**) forest gain agreement. The quantities represent the number of datasets that agreed in the direction of forest cover change among the four datasets. For each pixel, zero (white color) means no change was detected by any dataset whereas four means the change was detected by all four datasets. The stacked barplots in the lower right insets show the percentage of agreement of each dataset. For MODIS VCF and GFC, pixels with non-zero tree cover change were considered to be forest loss or gain. Maps were created using Python 2.7.11 (https://www.python.org/).

Forest loss estimates derived from different satellite datasets for the 2000s showed relatively high consistency in both spatial pattern and total area lost (Fig. 1), indicating the good agreement of satellite datasets in detecting forest loss. We introduced a quantitative indicator to evaluate the spatial agreement of forest cover change among different datasets, defined by the count of datasets in which a change was detected for a given pixel. Agreement levels 3–4 and 1–2 were considered as “high” and “low” agreement respectively, and level 4 indicated “full” agreement. Most forest loss was concentrated in south, southeast, and northeast China, which also corresponded to the regions with high spatial agreement across datasets (a change confirmed by at least 3 datasets, blue and red on the map in Fig. 2a). The estimates of total forest loss ranged from −0.56% to −0.69% of the total land area of China for three datasets (54,019 km^2^ in MODIS NBR, 65,948 km^2^ in VCF, and 61,165 km^2^ in GFC), while MODIS LC gave a larger loss of −1.58% (151,846 km^2^). The differences among forest loss estimates cannot be simply attributed to the difference in the time span of each dataset. As shown in the annual forest loss area available in GFC (Supplementary Fig. S2), the area of forest loss around 2001 and 2012 was rather low compared to other years, suggesting that the inclusion or exclusion of certain years could have some impacts. However, this observation is still not enough to fully explain the discrepancies found across datasets, especially for MODIS LC.

### Forest cover gain estimates from satellite datasets

**Figure 3:**
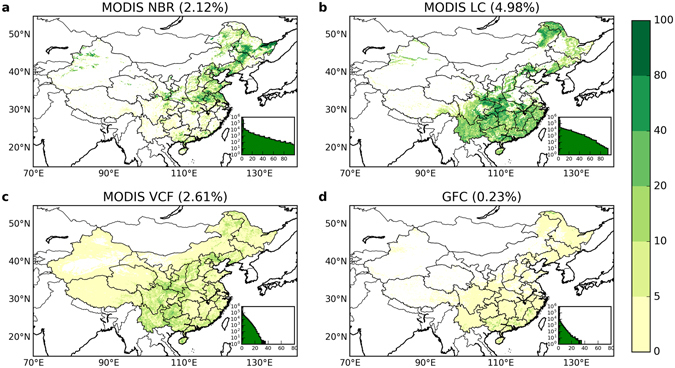
Spatial pattern of forest gain in China between 2000 and 2013 from different satellite datasets. These patterns were computed from similar (but not identical) time periods: (**a**) MODIS NBR for 2000–2013, (**b**) MODIS LC for 2001–2012, (**c**) MODIS VCF for 2000–2013, (**d**) Landsat GFC for 2000–2012. The total forest gain area was 207,639 km^2^ in MODIS NBR, 478,040 km^2^ in MODIS LC, 250,919 km^2^ in MODIS VCF, and 22,405 km^2^ in GFC. The other attributes of these panels were the same as for Fig. 1 but for forest gain. Maps were created using Python 2.7.11 (https://www.python.org/).

In contrast to forest loss, forest gain estimates showed large discrepancies across datasets in both the spatial pattern and the total change area (Fig. 3). Forest gains in the MODIS NBR were mainly observed in north and northeast China, but the MODIS LC showed a more widespread pattern throughout the northeast region and most parts of south China. The spatial patterns in forest gain for MODIS VCF and GFC were similar to MODIS LC. High agreement in forest gain was observed in central south China, and in some parts of northeast China (Fig. 2b). However, the numbers of forest gain pixels that highly agree were smaller than the numbers of those for forest loss in 3 out of 4 datasets. In addition, the total forest area gained had a much wider range (0.23% to 4.98%) than the total forest area lost (−0.56% to −0.69%, and −1.58% for MODIS NBR). The largest increase in forest area was found in MODIS LC, equal to 4.98% (478,040 km^2^) of the total land area of China, while the smallest increase is observed in GFC at merely 0.23% (22,405 km^2^). MODIS NBR (2.12%, 207,639 km^2^) and VCF (2.61%, 250,919 km^2^) showed similar overall gains despite different spatial patterns.

### Net forest cover change estimates from satellite data and forest inventory data

**Figure 4:**
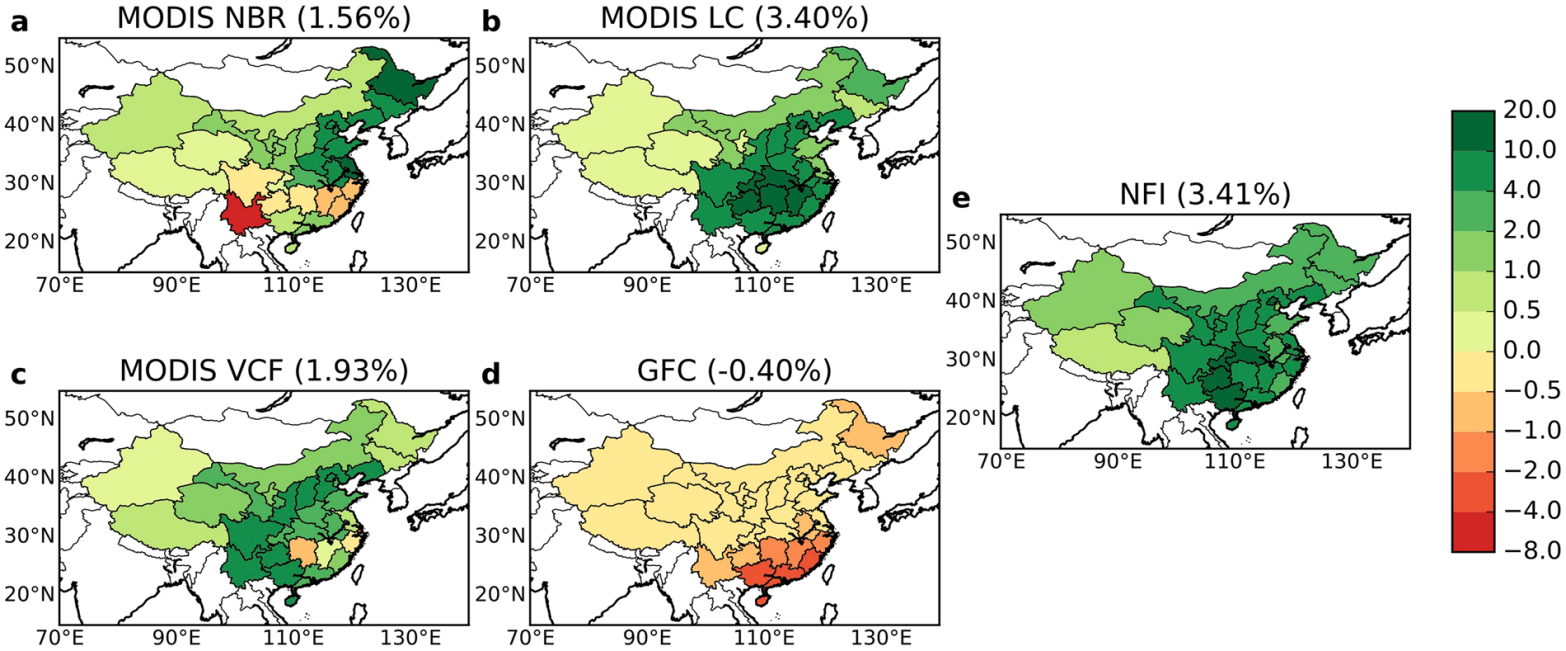
Net forest cover change from satellite data (**a**) MODIS NBR, (**b**) MODIS L﻿C, (**c**) MODIS VCF, (**d**) Landsat GFC, and (﻿**e**)the Chinese National Forest Inventory (NFI) data at the provincial level (%). Provincial forest change is presented as the area of change relative to the total area of each province (%). The number in parentheses next to the title shows the corresponding net change in national forest coverage. Maps were created using Python 2.7.11 (https://www.python.org/).

The net change in forest cover (forest gain minus forest loss) from satellite datasets was aggregated to the provincial level to compare with the NFI statistical dataset (Fig. 4). According to NFI, China’s forest area has increased from 1,749,100 km^2^ in the 6th survey (1999–2003) to 2,076,500 km^2^ in the 8th (2009–2013) survey, corresponding to a 3.41% increase in national forest coverage. Generally, MODIS-based datasets (LC, VCF, NBR) provided increase estimates similar to NFI in terms of provincial change. Interestingly, MODIS LC showed a similar change (3.40%) to NFI, whereas all other satellite datasets showed a smaller increase (1.56% for MODIS NBR and 2.93% for VCF). In contrast, a net decrease in forest cover was observed in GFC (−0.40%) and for all provinces. This is due to the lower forest gain estimates from the GFC dataset, while the forest loss estimate remained of similar magnitude to the other datasets.

**Figure 5:**
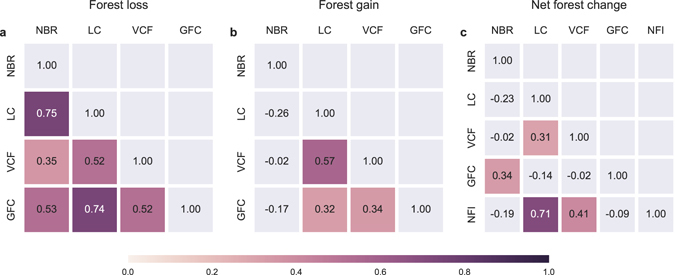
Correlations at provincial level of (**a**) forest loss, (**b**) forest gain, and (**c**) net forest change between the four satellite datasets and the statistical NFI data. Provincial forest change area was divided by the land area of each province to convert to forest coverage prior to the correlation calculation. Each box in the correlation matrix contains a value of Pearson correlation between two given datasets. The NFI data were only available for estimates of the net forest change. Insignificant correlations by *t*-test at 90% level are presented in grey color. A similar figure but for the weighted correlations (forest cover change area weighted by the amount of provincial forest area) is provided in Supplementary Fig. S3. The provincial statistics for forest loss, gain, and net change are provided in Supplementary Tables S2 to S4.

The pair-wise correlations in forest change between each two datasets provide a quantitative way to evaluate the consistency of provincial forest cover change estimates across these datasets (Fig. 5). More of these correlations were significant (*t*-test at 90%) for forest loss than for forest gain (6 for loss versus 3 for gain). In addition, correlation coefficients on average were higher for forest loss than for forest gain. A similar figure but displaying weighted correlations (forest change area weighted by the amount of forest area, Supplementary Fig. S3) shows even more polarized differences between forest loss and gain, indicating that forest loss had similar provicial-level spatial patterns between all four satellite datasets while there was very little agreement for forest gain. Specifically, forest gain in MODIS NBR was negatively correlated with the other three (Fig. 5b), while the correlations among the rest of the datasets (MODIS VCF, MODIS LC, and GFC) were all positive. There were only few significant correlations observed in net forest change, which was a consequence of the lack of significant correlations in forest gain.

### Causes of discrepancies in forest cover change estimates

It is challenging to identify the specific causes for the discrepancies in forest cover change estimates because the satellite products and the NFI statistics were developed using different sources, methodologies, and to serve different user communities. Here we focused on three key factors: algorithm parameter values^29^, spatial resolution^65, 66^, and forest definition^40, 67, 68^. These three factors are not exhaustive but demonstrate their substantial influences on the detection of forest cover change.

First, we used MODIS NBR as an example to investigate the sensitivity of forest cover change results to algorithm parameter values. A range of alternative values were tested for three parameters in MODIS NBR, (1) the forest NBR threshold to distinguish forest and non-forest (forest threshold hereafter), (2) the NBR threshold to determine change signal (change threshold hereafter), and (3) the forest mask to filter forest change signals. With higher NBR thresholds used to define forest or change (Supplementary Fig. S4), the amount of forest gain decreased from 3.89% to 0.80% and forest loss also decreased from 0.79% to 0.17%. With a forest mask defined by a higher tree cover threshold, forest land area decreased, which decreased the likelihood of forest loss while increasing the likelihood of forest gain (Supplementary Fig. S5). Although the amount of detected forest change varied greatly, forest gain was consistently higher than loss regardless of the choice of parameter value in MODIS NBR.

**Figure 6:**
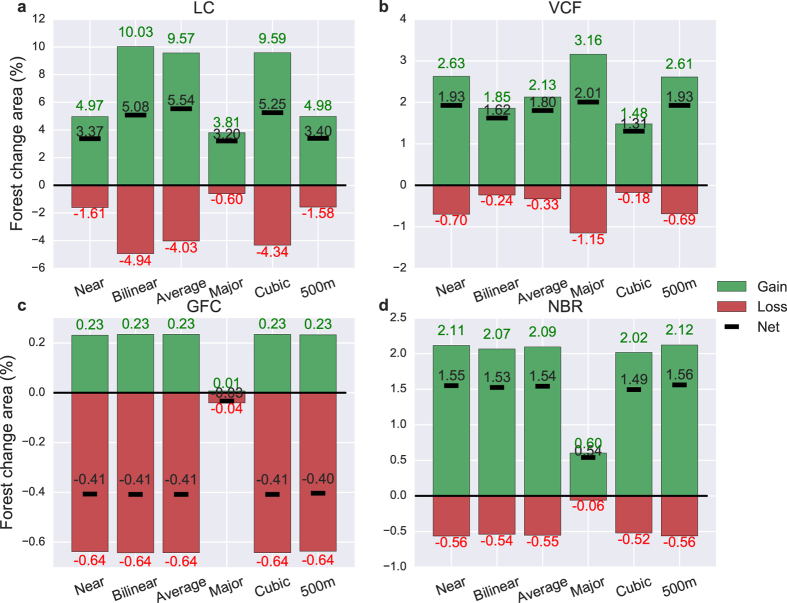
Forest cover change resampled from 500 m to 5 km spatial resolution for ﻿(**a**) MODIS LC, (**b**) MODIS V﻿CF, (**c**) GFC, and (**d**) MODIS NBR using different resampling methods. Five different resampling methods were applied to emulate the range in spatial scaling processes available. These include nearest neighbor (near), bilinear, average, mode (major), and cubic from the Geospatial Data Abstraction Library. These resampled forest change estimates at 5 km were compared against the original forest change area at 500 m.

Second, to demonstrate the influence of spatial resolution, we resampled the satellite datasets from 500 m to 5 km resolution using different methods and compared the resampled forest cover change results with the original 500 m results. For MODIS LC and VCF data, we first resampled the forest maps of two periods (2001–2003 and 2010–2012) to 5 km and then extracted forest cover change from the resampled maps. This method is similar to how resolution change would affect satellite images in the real world. We found that changing spatial resolution (scaling) has noticeable impacts on forest cover change detection (Fig. 6). MODIS LC and VCF were most affected by resolution changes. Forest gain (loss) area varied from 3.81–10.03% (0.6–4.94%) in MODIS LC and from 1.85–3.16% (0.24–1.15%) in VCF. Note that the bilinear, average, and cubic resampling methods are not suitable for categorical MODIS LC data. For MODIS NBR and GFC, we resampled the change maps instead of the forest maps, because these two datasets directly provided change estimates at their original resolution. We found that the amount of change in these two datasets was least affected by resampling (except for the majority resampling method because the number of changed pixels at 500 m were too few relative to the 5 km pixel size). This is understandable because in this case resampling had no impact on the source data at which forest cover change in MODIS NBR and GFC was extracted.

**Figure 7:**
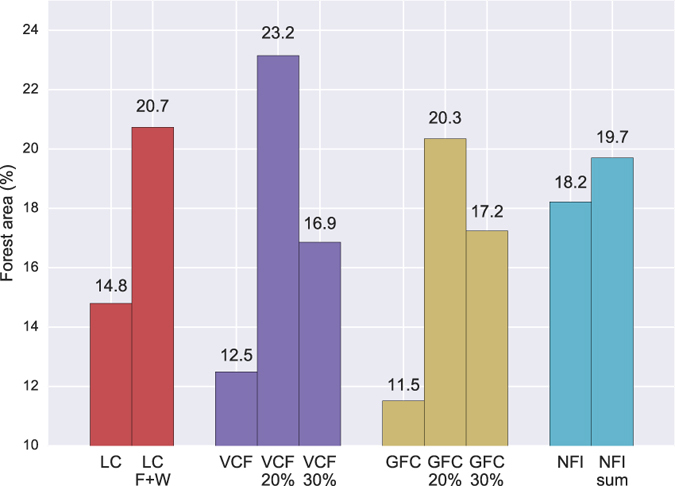
The total forest area in China with different forest definitions at the beginning of 2000s. The number on top of each bar is the total forest area represented as national forest coverage (%). For MODIS LC, forest was defined by the five IGBP forest land cover types in the product, and woody savanna was also included as a modified definition (LC F + W). For MODIS VCF and GFC, forest was defined by fractional tree cover (VCF, GFC) or by the binary tree cover using the 20% (VCF 20%, GFC 20%) and 30% (VCF 30%, GFC 30%) thresholds. Forest area was calculated as the areal sum of all non-zero fractional tree cover (numeric) in MODIS VCF and GFC, or the areal sum of forest pixels in MODIS LC and in the binary VCF and GFC. For NFI, forest was defined as woodland with canopy coverage >20%. The total forest area was the value reported in the 6^th^ NFI. The NFI sum gives the total forest area summed up from the provincial numbers in the 6^th^ NFI.

Third, we investigated the potential influence of forest definition on forest cover change results by comparing total forest area at the beginning of the study period across different datasets using default and modified forest definitions (Fig. 7). The modified forest definition for MODIS LC (LC F + W) included the original forest classes as well as woody savanna, which contains forest canopy cover between 30% and 60%^61^. The modified forest definitions were also applied to VCF and GFC, by converting their original definition of fractional tree cover to a binary forest/non-forest variable using 20% (VCF 20% and GFC 20%) and 30% (VCF 30% and GFC 30%) thresholds. For the NFI data, we noticed that the summed provincial forest area (NFI sum) was not the same as the reported national value. Their difference (1.5%) was indeed quite large compared to the reported forest increase of 3.41%, thus both statistics were included. Results showed that the total forest area varied considerably across different datasets and across different definitions (from 11.5% in GFC to 23.2% in VCF 20%, Fig. 7). The forest area in satellite data with the default forest definitions (11.5–14.8%) was generally lower than that in the NFI data (18.2%), while the area was higher when modified forest definitions were used. The larger forest area with the modified definition for MODIS LC (LC F + W) was due to the inclusion of woody savanna, and for the binary VCF and GFC maps was due to forest area being calculated by the total size of forested pixels instead of by sub-pixel fractional cover. It appears that the different forest area estimates created by different forest definitions alone had a magnitude comparable to, or larger than^69^, the amount of actual forest cover change, which could potentially influence forest cover change estimate in a similar way to the forest mask in MODIS NBR (Supplementary Fig. S5).

## Discussion

Our results suggest that estimates of forest gain were the major factor driving the differences in the net forest cover change observed among the four satellite datasets and the NFI statistical data during the 2000s (2000–2013). In most datasets (except for GFC) the net change in forest cover was dominated by forest gain, therefore, the large discrepancies in the magnitude of net change were primarily consequences of the poor agreement in forest gain (Fig. 5). Although the datasets had better agreement in estimates of forest loss, the total magnitude of this change was much smaller on average and was not enough to compensate the substantial variance in the estimates of forest gain. The lack of consistency among forest gain estimates could reflect the fact that forest growth is a more gradual^24^ and complex ecological process, with many characteristics that vary depending on tree species, management, and climate. This gradual signal is more difficult to detect than forest loss^70, 71^, which is often an abrupt signal that happens in a short time span (note that certain types of forest loss could be a gradual process, e.g., loss due to insects^58^). The lower detectability of forest gain can be seen from the accuracy assessment of remotely sensed forest cover change products, in which forest gain usually has a lower accuracy than forest loss^23, 33, 70^. For example, in the GFC product^33^, the accuracy of forest gain detection was lower than forest loss (73.9–76.4% vs. 87.0–87.8%) at the global scale, and the difference was even larger in temperate regions (62.0–76.5% vs. 88.2–93.9%), where China is located. The environment^70^ where forest cover change takes place can also influence its detectability. Detecting forest growth could be additionally complicated by an overall increase in vegetation productivity in these regions induced by climate change^72^, ecosystem restoration^73^, and non-forest vegetation recovery after disturbance, which could provide false positives in some measurements.

There are several additional factors that could have contributed to the differences in forest cover change estimates from satellite data. These include the sensitivity of forest change results to parameter values^29^, spatial resolution^65, 66^, and forest definition^40, 67, 68^. We found that by changing parameter values (e.g., the threshold to determine forest loss and gain) in the MODIS NBR analysis, the amount of detected forest change varied greatly. Interestingly, forest gain was always higher than loss regardless of the choice of parameter value (Supplementary Figs S4 and S5). This emphasizes the importance of parameter calibration and training with reliable reference datasets^23^ for the detection algorithm. Similarly, depending on the patch size^66, 74^ of forest cover change, its detectability can be greatly affected by the difference in spatial resolution because small, sub-pixel changes, which are common in these regions, cannot always be captured by data from a coarse resolution sensor^23^. Therefore, the amount of change “seen” by satellite data at different resolutions can be quite variable^75^ due to information loss with reduced spatial resolution (Fig. 6). The factors investigated here can produce uncertainty in the detection of forest change of a similar or larger magnitude than the real change signal, which can be subtle in nature.

Perhaps the most important factor driving the inconsistent estimates of forest change across datasets was the various definitions for “forest”^34, 40, 69, 76^ and “forest change”^39, 41, 77–79^. Some special land cover types included by the NFI forest definition in China (e.g., special shrubs^64^) may not necessarily be defined as forest in remote sensing. Definitions of forest in remote sensing and NFI datasets can lead to discrepancies in forest area far exceeding the actual change signal (Fig. 7). Similarly, the inconsistent definition for “forest change” between remote sensing and forest inventory datasets weakens comparability of forest cover change across datasets^41, 78^. For example, the high frequency sampling (days to weeks) of remote sensing observations captures changes in forest cover such as temporary forest loss due to disturbance or harvest followed by slow regrowth, particularly in MODIS NBR and GFC in which forest cover change is detected by spectral trend analysis. These changes, however, do not usually result in a change in land use and, therefore, may not be captured by forest inventory^77^ or from differences in land cover between two end dates (e.g., MODIS LC and VCF). Such compatibility issues can also appear in remote sensing due to their different data processing procedures and change detection methods (spectral based such as NBR and GFC vs. thematic based such as MODIS LC and VCF)^22, 59, 79^. It is still unclear how much contribution each of these two types of changes (temporary vs. permanent) had on the detected forest loss in our analysis. Distinguishing these context-dependent terminologies and reconciling their inconsistences can improve the characterization of forests^40, 68^ and the accuracy of forest change monitoring^41, 67, 69^.

Due to the large discrepancies in forest cover change estimated by these four different satellite datasets, it is hard to directly verify the claim of 3.41% increase in forest coverage in China because that quantity is still within the variation across these datasets. Although most of the estimates (except GFC) showed a net increase in forest cover (from 1.5% to 3.4%), there are other studies that have shown a net decrease in forest cover. For example, another Landsat-based forest cover change map, produced by the Global Land Cover Facility, indicated forest loss outweighed forest gain in China between 2000 and 2005^80^. According to existing evidence, it seems likely that estimates from the coarse resolution data such as MODIS are more likely to support forest cover increase whereas Landsat-based datasets, presumably more accurate due to their high spatial resolution^66, 79, 81^, provide more inconsistent results. These estimates from Landsat data indicated either a net decrease (see ref. 80 and −0.4% in GFC^33^) or a very small net increase (0.02% in ref. 82) in forest cover. This result suggests another possibility that China’s forest cover could have stabilized during the 2000s (2000–2013), but further studies are needed to verify this hypothesis.

## Conclusion

Our results show varying estimates of forest cover change in China from 2000 to 2013 from multiple satellite and forest inventory datasets, suggesting large uncertainty in the direction and magnitude of the net forest change during this period. The forest area increase claimed by the National Forest Inventory (NFI) data cannot be directly verified by satellite data because of the mixed results caused by different definitions and methodologies, although it is in agreement with certain MODIS dataset (i.e., MODIS LC). The inconsistent estimates of forest cover change from satellite data could be caused by factors such as algorithm parameters, spatial resolutions of monitoring, and forest definition, all of which can strongly influence the estimates with a magnitude comparable to the real change signal. Considering the increase in forest cover indicated by the majority of datasets, combined with independent evidence from other studies^47, 83–85^ and the implementation of forest conservation policies^21, 42–45, 86^, a large decrease in China’s forest cover during the study period is less probable. However, we cannot rule out the possibility that forest cover for this period was relatively stable with comparable magnitudes of forest loss and gain. Thus, due to the internal uncertainties and methodological differences, using one of these satellite datasets or the NFI data to deduce the “real” forest change must be undertaken with caution. To obtain accurate estimates of forest change, efforts must be made to validate and assess satellite data using ground-level field data, to improve methods for detecting forest gain, and to reconcile inconsistencies in definitions of forest and forest change using methods such as data fusion^67, 87–89^.

## Electronic supplementary material

Supplementary Information
