# Supplementary material for: Inconsistent estimates of forest cover change in China between 2000 and 2013 from multiple datasets: differences in parameters, spatial resolution, and definitions
Source: Sci Rep. 2017 Aug 18;7:8748. doi: 10.1038/s41598-017-07732-5 (PMC5562877; doi:10.1038/s41598-017-07732-5)
Supplement: Supplementary file 1 — Supplementary Information [file 41598_2017_7732_MOESM1_ESM.pdf]

Supplementary Information for Inconsistent estimates of forest cover change in China between 2000 and 2013 from multiple datasets

Yan Li[1,2,3], Damien Sulla-Menashe[4], Safa Motesharrei[2,5], Xiao-Peng Song[6], Eugenia Kalnay[1,2], Qing Ying[6], Shuangcheng Li[3,7], Zongwen Ma[8]

1 Department of Atmospheric and Oceanic Science, University of Maryland, College Park, Maryland 20742, USA.

2 The Institute for Physical Science and Technology, University of Maryland, College Park, Maryland 20742, USA.

3 College of Urban and Environmental Sciences, Peking University, Beijing 100871, China.

4 Department of Earth and Environment, Boston University, Boston MA 02215, USA

5 Department of Physics, University of Maryland, College Park, Maryland, 20742, USA.

6 Department of Geographical Sciences, University of Maryland, College Park, Maryland 20742, USA.

7 Key Laboratory for Earth Surface Processes of The Ministry of Education, Peking University, Beijing 100871, China.

8 China Science and Technology Exchange Center, Beijing 100045, China.

Correspondence: Yan Li, yanli.geo@gmail.com

Forest cover change estimate using MODIS NBR time-series

The MODTrendr algorithm

The MODTrendr algorithm was adapted from LandTrendr [1] to detect forest disturbance and recovery with time-series of MODIS surface reflectance data [2] and was used for this purpose in this study. To perform the temporal segmentation, piece-wise linear regressions are applied to time series of annual, peak growing season spectral observations. The residual errors of each regression are compared and potential vertices can be identified which define the start and end points of each segment. This is a recursive process until a significance level is reached. The strength of this strategy is that no *a priori* information about the surface is required and it can be applied to any spectral quantity or index.

For this study, a time series of the Normalized Burn Ratio (NBR) was used as input the MODTrendr algorithm (referred as MODIS NBR hereafter). NBR was calculated using near infrared (NIR; MODIS band 2) and short-wave infrared (SWIR; MODIS band 7) reflectance values from MODIS: NBR = (NIR − SWIR)/(NIR + SWIR) [5]. The NBR is a unitless index that typically ranges from 0 to 1. Note the NBR value used in the study was multiplied by 1000 to have a range from 0 to 1000 for ease of interpretation. At each MODIS pixel, an annual time series of NBR from 2000 to 2013 at 500 m was constructed from MODIS surface reflectance data. First, for each year (2000-2013) we extracted all available observations between July and mid-August (day of year 180–220) from the Nadir BRDF-Adjusted Reflectance (NBAR) product (MCD43A4), and then computed a quality-weighted arithmetic mean based on the associated quality flags provided by MODIS NBAR Quality Assurance product (MCD43A2). The resulting late-summer reflectance composites were then used to

calculate the NBR for each year and these NBR values were used to perform segmentation with MODTrendr.

The outputs of MODTrendr contain specific information about the NBR value and year of each identified vertex (two vertices determine one segment) in the time series. Forest change information can be derived from the slope and length (in years) of the segments derived from the NBR time series. In principle, a disturbance event corresponds to a segment with negative slope and growth corresponds to a segment with positive slope. The length of the segment defines the disturbance and growth duration and the difference in NBR between two vertices is used to characterize the magnitude of change. Because our time series were 14 years in length, the maximum number of segments was set to 4. The lack of ground validation reduces our confidence in the MODTrendr results because its parameter values were not fully optimized for detecting forest change in China. To compensate for this, we carried out an additional sensitivity analysis to investigate the impact of parameter values on the detection of forest change.

Forest NBR threshold and NBR forest change threshold

In order to extract forest change based on the MODTrendr outputs, a forest NBR threshold was needed to separate forest from non-forest and another change threshold was needed to differentiate the real change signal from noise. To determine the optimal threshold in NBR to distinguish between forest and non-forest, we intersected annual late-summer NBR values with the stable land cover map of MODIS LC product (MCD12Q1[6]) from 2001 to 2005 and then averaged the NBR values over different forest and non-forest types (Supplementary Table S1). We found that NBR values for most forest types were consistently higher than 600 with the exception of deciduous needleleaf forest (550 in 2001). In contrast, NBR values for non-forest

types were typically lower than 500 with the exception of woody savannas (506 in 2001), permanent wetlands (588 in 2001), cropland/natural vegetation mosaic (519 in 2001), and snow and ice (554). For wetland and snow, their high NBR values were due to the confounding effect of water. These classes were later masked out using the stable land cover map. The high NBR values observed in woody savannas and cropland/natural vegetation mosaic are not surprising because by definition they are mixed ecosystems that contain forested land [7]. Given that there is no definitive criterion to distinguish forest from non-forest and an overlap between these two classes is common for both remote sensing and NFI, here we chose the lowest NBR value among forest types (550, deciduous needleleaf forests), as the threshold to distinguish forest and non-forest types. With this threshold, some pixels originally classified as woody savanna, and cropland/natural vegetation mosaics would be considered as forest instead of non-forest for forest cover change detection. It is likely that forest gain detection in regions where significant changes in these classes have occurred would be less accurate and prone to error (e.g., the Northeast and Central-North agricultural regions).

A segment containing a change in NBR could either reflect a real change signal or noise in the reflectance data. A real forest change signal would likely result in a large (negative) change in NBR, while small change in NBR would be more likely to be noise. We calculated the standard deviation (SD) in NBR among all forest cover types (70) and chose 2 × SD (140) to be the NBR threshold that differentiates the real forest change signal from noise, because normally about 95% forests fall in this NBR range (Supplementary Table S1). Therefore, a change exceeding this threshold means that the pixel has undergone forest cover change.

Forest gain and loss detection

Based on the MODTrendr outputs, potential forest loss or gain signals can be extracted from MODIS pixels experiencing disturbance (segments with negative slope) or growth signals (segments with positive slope). The greatest disturbance (the largest NBR decline) event is considered to be forest cover loss when the following conditions are satisfied: (1) NBR drops from the above NBR forest threshold to below the forest threshold for the disturbance event and (2) the magnitude of NBR change is larger than the forest change threshold. Three types of forest cover loss signals were identified in the NBR time series including monotonous decline, severe disturbance, and disturbance followed by a recovery (Supplementary Figure S1a-c).

Forest gain, unlike the abrupt change shown by disturbance segments, is a slow growth process that may last for years and is therefore more difficult to detect. In theory, without disturbance, forest growth should manifest as a gradual increase in NBR. With this knowledge, forest gain can be identified when the following conditions are satisfied: (1) one or more consecutive segments show continuously increasing NBR until the end of study period; (2) the NBR increases from below the forest NBR threshold to above the forest NBR threshold; and (3) the cumulative NBR increase is larger than the forest change threshold. It should be emphasized that the presence of forest growth signals could be caused by afforestation, natural growth, or recovery after disturbance. In order to minimize the confounding effect of recovery from disturbance, pixels identified to experience forest cover loss were removed in the forest gain detection, while small disturbances that were not considered as a forest loss signal were retained. Even with these rules, the influence of disturbance on forest growth detection still exists to some extent. For example, recovery from a disturbance event that occurred prior to 2000 would be indistinguishable from the actual growth

signal from afforestation after 2000. Different types of forest gain in NBR time-series are shown in Supplementary Fig. S1d-f, including monotonous increases and growth following a minor disturbance event.

Potential forest loss signals as described above could appear in non-forest pixels, or gain signals could appear in forest pixels. To avoid these situations, we created a forest mask to filter out these unwanted pixels. The forest mask was created from the GFC tree cover map by using the 20% threshold, consistent with the forest definition adopted by the Chinese NFIs (canopy coverage >20%). The purpose of this filtering process is to make sure NBR forest loss signals only appear on forested and NBR forest gain signals only appear on non-forest pixels. Forest gain and loss results after this filtering were used for the analysis.

Supplementary Table S1. NBR values for different forest and non-forest land cover types in China (mean ± 2 × SD). Pixels with negative NBR value were excluded when computing means.

Land type	2001	2002	2003	2004	2005
1Evergreen Needleleaf	602±80	603±80	599±83	597±86	597±85
2Evergreen Broadleaf	643±47	643±46	642±45	642±44	642±44
3 Deciduous Needleleaf	550±64	551±63	543±71	547±67	551±64
4 Deciduous Broadleaf	663±128	664±129	663±131	664±131	663±131
5 Mixed forest	635±69	637±68	634±72	634±71	635±70
All forest	630±70	631±69	628±73	629±71	630±70
6 Closed shrublands	459±138	472±139	481±140	487±141	492±142
7 Open shrublands	365±174	372±175	375±175	379±175	383±176
8 Woody savannas	506±153	507±153	504±155	506±153	508±153
9 Savannas	430±104	440±100	444±100	454±97	463±94
10 Grasslands	193±154	196±155	195±154	196±154	196±154
11 Permanent wetlands	588±89	589±89	585±87	587±86	588±85
12 Croplands	316±177	318±177	313±179	314±177	315±178
13 Urban and built-up	281±155	281±153	279±154	278±154	278±154
14 Cropland/Natural vegetation mosaic	519±136	522±137	522±137	524±136	527±136
15 Snow and ice	554±222	559±220	564±219	566±218	567±218
16 Barren or sparsely vegetated	99±120	102±121	104±123	106±125	108±127
All Non-forest	295±201	297±201	296±201	297±201	298±202

Supplementary Table S2. Forest loss summary statistics (km^2).

	MODIS NBR	MODIS LC	MODIS VCF	GFC
Anhui	527	1699	913	1193
Beijing	6	809	48	20
Chongqing	212	2251	1496	132
Fujian	2466	7045	3012	7106
Gansu	73	804	164	124
Guangdong	2970	8585	4129	9001
Guangxi	3193	14311	3579	12330
Guizhou	1579	10267	905	1683
Hainan	518	2154	560	1472
Hebei	59	1861	452	338
Heilongjiang	3822	13517	8328	4909
Henan	107	732	1140	204
Hubei	477	2881	2242	643
Hunan	1568	8986	6237	4086
Jiangsu	45	1468	1683	53
Jiangxi	2768	7711	3827	4419
Jilin	296	3412	3069	623
Liaoning	80	2129	728	759
Nei Mongol	3425	9009	4662	2617
Ningxia	0	21	32	2
Qinghai	8	187	470	10
Shaanxi	115	1485	437	230
Shandong	15	117	337	47
Shanghai	0	79	246	0
Shanxi	110	2456	447	475
Sichuan	5074	11965	4362	1345
Tianjin	2	56	38	12
Xinjiang	746	977	2573	115
Xizang	1091	3583	2179	244
Yunnan	21421	27184	5012	5265
Zhejiang	1245	4108	2641	1703
Total	54019	151846	65948	61165

Supplementary Table S3. Forest gain summary statistics (km^2).

	MODIS NBR	MODIS LC	MODIS VCF	GFC
Anhui	10925	10595	5738	301
Beijing	761	2838	790	1
Chongqing	429	17499	3459	71
Fujian	1399	15235	4735	2868
Gansu	5651	8164	11188	12
Guangdong	4945	20742	8185	3919
Guangxi	4835	29475	14331	5346
Guizhou	1369	30096	14597	723
Hainan	835	2172	2374	584
Hebei	12905	12473	9372	60
Heilongjiang	51180	25174	12099	2079
Henan	12266	7509	5178	39
Hubei	4667	31885	7068	128
Hunan	939	30941	4721	898
Jiangsu	10319	2875	2384	12
Jiangxi	1828	25107	4220	1369
Jilin	15398	5300	4188	345
Liaoning	13986	10008	6940	150
Nei Mongol	13976	31782	19402	545
Ningxia	588	190	1042	0
Qinghai	988	481	9398	2
Shaanxi	4164	21169	14320	60
Shandong	11683	1929	4649	19
Shanghai	92	92	79	0
Shanxi	2830	8940	8162	15
Sichuan	4622	51197	26422	314
Tianjin	979	483	415	1
Xinjiang	10408	5676	5915	11
Xizang	1109	6328	12448	26
Yunnan	1257	51248	24575	2197
Zhejiang	306	10435	2524	306
Total	207639	478040	250919	22405

Supplementary Table S4. Net forest cover change summary statistics (km^2). The last column is the forest area in the 6th NFI, which is used to calculate the area weighted correlation for Supplementary Fig. S3. Note that in NFI the sum of provincial change area is not equal to the reported national value.

	MODIS NBR	MODIS LC	MODIS VCF	GFC	NFI	6th NFI
Anhui	10397	8896	4824	-892	4843	22572
Beijing	754	2029	742	-19	2093	9755
Chongqing	217	15249	1963	-62	13326	62110
Fujian	-1067	8190	1723	-4238	3633	16933
Gansu	5578	7360	11024	-112	20782	96861
Guangdong	1975	12158	4056	-5082	7913	36881
Guangxi	1642	15164	10752	-6984	35887	167260
Guizhou	-209	19829	13691	-960	23288	108540
Hainan	317	18	1814	-888	2111	9839
Hebei	12847	10612	8919	-278	11050	51502
Heilongjiang	47356	11657	3771	-2829	16463	76731
Henan	12159	6777	4038	-165	8877	41374
Hubei	4190	29003	4827	-516	23431	109210
Hunan	-628	21955	-1516	-3188	15115	70448
Jiangsu	10274	1408	702	-41	8469	39473
Jiangxi	-940	17395	393	-3050	7042	32822
Jilin	15102	1887	1119	-278	4375	20391
Liaoning	13907	7879	6212	-609	7678	35786
Nei Mongol	10551	22773	14740	-2072	43723	203790
Ningxia	588	169	1010	-2	2144	9993
Qinghai	980	295	8928	-8	8919	41570
Shaanxi	4048	19684	13883	-170	18285	85223
Shandong	11668	1813	4312	-28	4996	23285
Shanghai	92	13	-167	0	492	2293
Shanxi	2719	6484	7716	-460	7422	34593
Sichuan	-452	39231	22060	-1031	23940	111580
Tianjin	977	427	377	-11	181	844
Xinjiang	9662	4699	3342	-104	21418	99826
Xizang	19	2746	10269	-218	8195	38195
Yunnan	-20164	24062	19564	-3067	35417	165070
Zhejiang	-939	6327	-118	-1396	4744	22111
Total	153619	326191	184971	-38760	396252	1846861

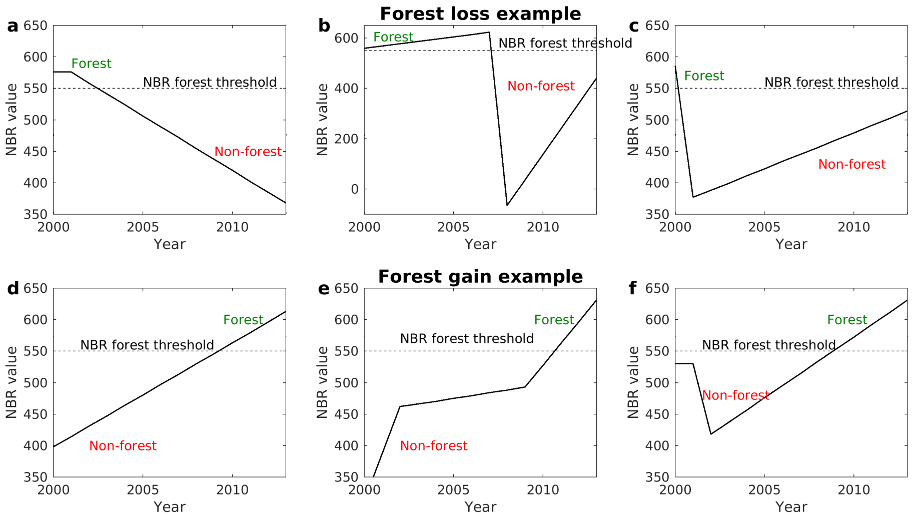

Supplementary Figure S1. Examples of forest cover change detected by NBR segments. Panels a-c show forest loss examples and panels d-f show forest gain examples.

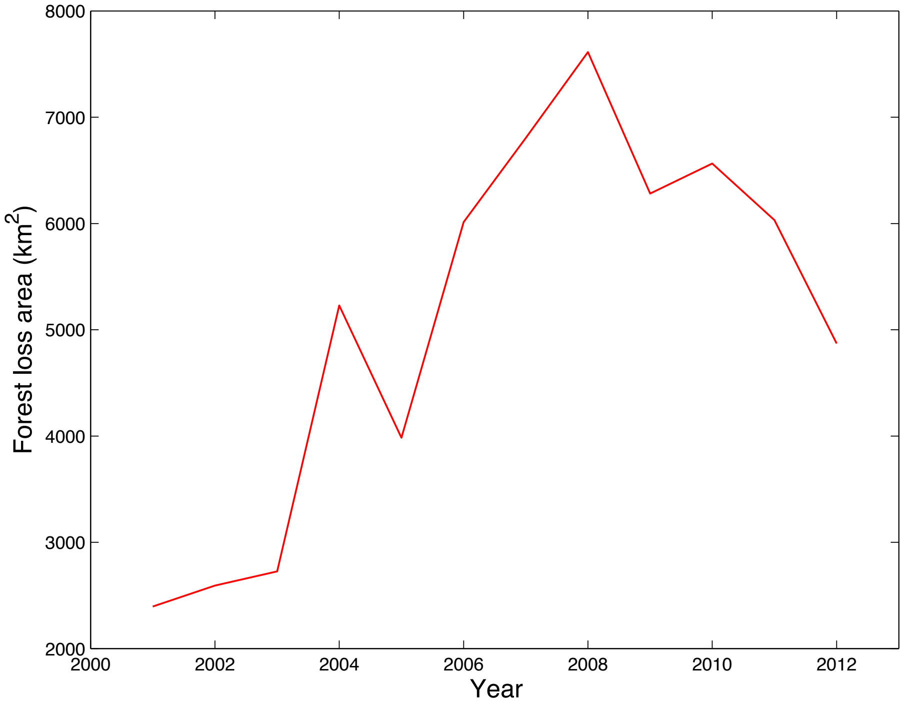

Supplementary Figure S2. Annual forest loss area in GFC data from 2000 to 2012 in China.

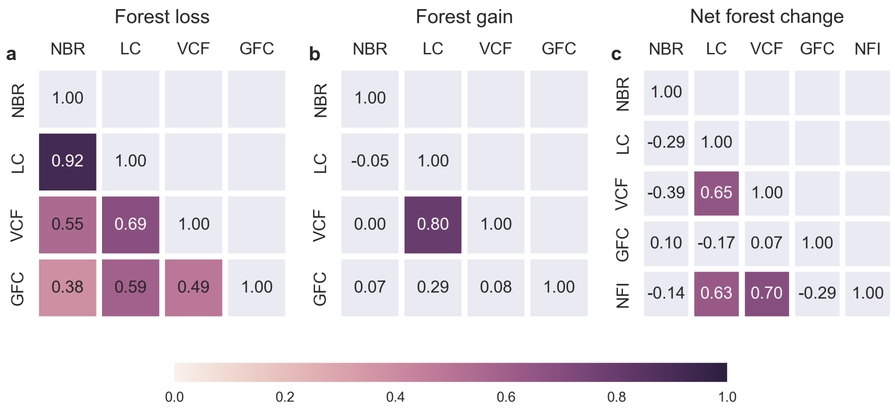

Supplementary Figure S3. Weighted correlations at provincial level of (a) forest loss, (b) forest gain, and (c) net forest change between four satellite datasets and the statistical NFI data. This figure differs to Fig.4 as it shows the weighted correlations. The provincial forest change area was weighted by the amount of forest area from the 6th NFI. Insignificant correlations by t-test at 90% level are colored in grey.

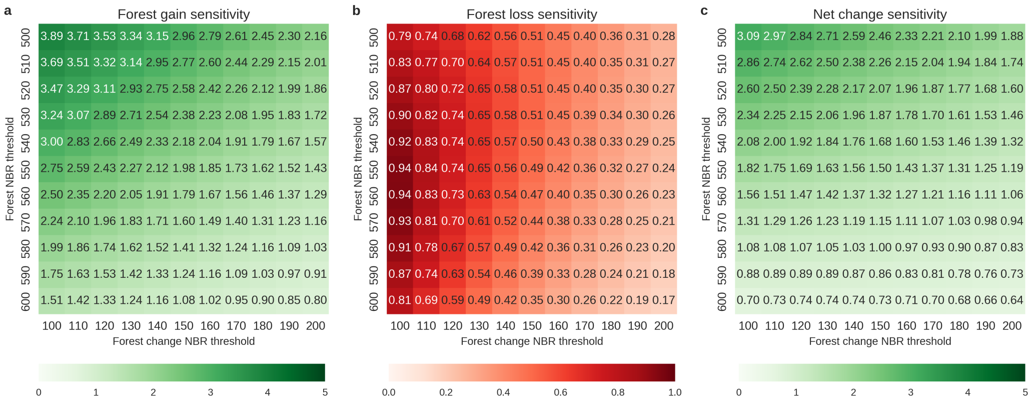

Supplementary Figure S4. Sensitivity of total change area of (a) forest gain, (b) forest loss, and (c) net forest change in China to NBR forest threshold (y-axis) and forst change threshold (x-axis) for MODIS NBR. Each value represents the total area of forest cover change in China under different threshold combinations, represented by a percentage to the country area of 9.6 million km^2. The forest NBR threshold is used to define whether a pixel is forest or non-forest based on its NBR value. The NBR forest change threshold is used to define whether a change is recorded as a possible forest change signal. Results reported in the main text are based on the selected NBR forest threshold of 550 and NBR forest change threshold of 140.

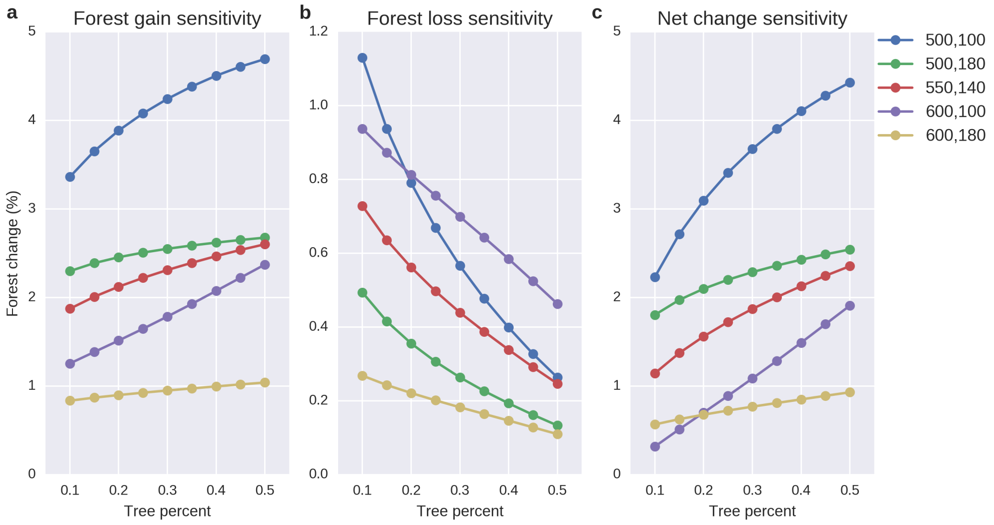

Supplementary Figure S5. The sensitivity of total change area of (a) forest gain, (b) forest loss, (c) and net forest change in China to forest masks created from different tree cover thresholds for MODIS NBR. Forest cover change is shown as a percentage to the country area of 9.6 million km^2. Each line represents the forest cover change under different combinations of forest NBR threshold and NBR forest change threshold. The forest mask is used to determine whether a loss signal corresponds to forest loss, or a gain signal corresponds to forest gain.

References:

1. Kennedy, R. E., Yang, Z. & Cohen, W. B. Detecting trends in forest disturbance and recovery using yearly Landsat time series: 1. LandTrendr — Temporal segmentation algorithms. *Remote Sens. Environ.* **114,** 2897–2910 (2010).
2. Sulla-Menashe, D. *et al.* Detecting forest disturbance in the Pacific Northwest from MODIS time series using temporal segmentation. *Remote Sens. Environ.* **151,** 114–123 (2014).
3. Jin, S. *et al.* A comprehensive change detection method for updating the National Land Cover Database to circa 2011. *Remote Sens. Environ.* **132,** 159–175 (2013).
4. Cohen, W. B., Yang, Z. & Kennedy, R. Detecting trends in forest disturbance and recovery using yearly Landsat time series: 2. TimeSync - Tools for calibration and validation. *Remote Sens. Environ.* **114,** 2911–2924 (2010).
5. García, M. J. L. & Caselles, V. Mapping burns and natural reforestation using thematic Mapper data. *Geocarto Int.* **6,** 31–37 (1991).
6. Friedl, M. A. *et al.* MODIS Collection 5 global land cover: Algorithm refinements and characterization of new datasets. *Remote Sens. Environ.* **114,** 168–182 (2010).
7. Friedl, M. A. *et al.* Global land cover mapping from MODIS: algorithms and early results. *Remote Sens. Environ.* **83,** 287–302 (2002).
